# Sequencing of Australian wild rice genomes reveals ancestral relationships with domesticated rice

**DOI:** 10.1111/pbi.12674

**Published:** 2017-01-23

**Authors:** Marta Brozynska, Dario Copetti, Agnelo Furtado, Rod A. Wing, Darren Crayn, Glen Fox, Ryuji Ishikawa, Robert J. Henry

**Affiliations:** ^1^ Queensland Alliance for Agriculture and Food InnovationUniversity of QueenslandBrisbaneQLDAustralia; ^2^ Arizona Genomics InstituteSchool of Plant SciencesUniversity of ArizonaTucsonAZUSA; ^3^ International Rice Research InstituteT.T. Chang Genetic Resources CenterLos BañosLagunaPhilippines; ^4^ Australian Tropical HerbariumJames Cook UniversityCairnsQLDAustralia; ^5^ Queensland Alliance for Agriculture and Food InnovationUniversity of QueenslandToowoombaQLDAustralia; ^6^ Faculty of Agriculture and Life ScienceHirosaki UniversityHirosakiAomoriJapan

**Keywords:** assembly, molecular clock, sequencing, *Oryza*, phylogeny, wild rice

## Abstract

The related A genome species of the *Oryza* genus are the effective gene pool for rice. Here, we report draft genomes for two Australian wild A genome taxa: *O. rufipogon*‐like population, referred to as Taxon A, and *O. meridionalis*‐like population, referred to as Taxon B. These two taxa were sequenced and assembled by integration of short‐ and long‐read next‐generation sequencing (NGS) data to create a genomic platform for a wider rice gene pool. Here, we report that, despite the distinct chloroplast genome, the nuclear genome of the Australian Taxon A has a sequence that is much closer to that of domesticated rice (*O. sativa*) than to the other Australian wild populations. Analysis of 4643 genes in the A genome clade showed that the Australian annual, *O. meridionalis*, and related perennial taxa have the most divergent (around 3 million years) genome sequences relative to domesticated rice. A test for admixture showed possible introgression into the Australian Taxon A (diverged around 1.6 million years ago) especially from the wild *indica*/*O. nivara* clade in Asia. These results demonstrate that northern Australia may be the centre of diversity of the A genome *Oryza* and suggest the possibility that this might also be the centre of origin of this group and represent an important resource for rice improvement.

## Introduction

Rice is a pantropical crop that is a staple food consumed by over half of the world's population This crop has a long history of domestication and its cultivation dates back around 10 000 years in Asia (*O. sativa*) and over 3000 years in Africa (*O. glaberrima*). The *Oryza* genus diversified into six diploid (A–C and E–G) and five tetraploid (BC, CD, HJ, HK and KL) genome groups. The phylogeny of the *Oryza* genome groups has been widely studied and is now well known (Ammiraju *et al*., 2010; Ge *et al*., 1999; Lu *et al*., 2009). The relationships between the most recently diverged A genome diploids, that include domesticated rice, have been more challenging and only lately have their phylogeny been more fully described, using both chloroplast (Wambugu *et al*., 2015) and nuclear genomes [International Oryza Map Alignment Project (I‐OMAP), unpublished].

**Figure 1:**
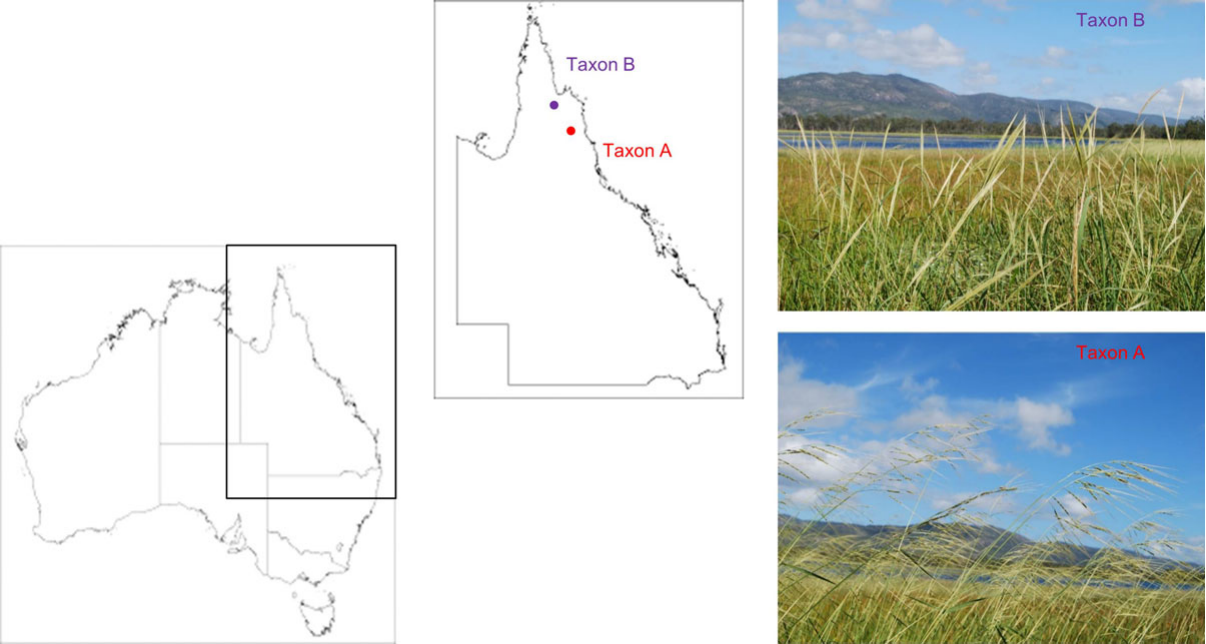
Australian perennial A genome taxa from northern Australia. Taxon A is characterized by open panicles, while Taxon B has closed panicles.

Rice food security requires continued increases in rice productivity and relies on ongoing genetic improvement. Climate change adds to the difficulty of achieving the necessary rates of genetic gain (Abberton *et al*., 2016). The wild relatives of rice provide a gene pool that allows for the expansion of diversity (Krishnan *et al*., 2014) in domesticated rice for the creation of new high yielding genotypes, with new nutritional and functional traits (Kharabian‐Masouleh *et al*., 2012) and adaptation to new environments (Brozynska *et al*., 2016). The A genome species of *Oryza*, which include the species that are readily interfertile with rice, represent the effective primary gene pool for rice. Recent investigations of large and widespread wild populations in tropical Australia (Henry *et al*., 2010) suggest the presence of two distinct and possibly novel perennial wild A genome taxa [Figure 1; Waters *et al*. (2012); Sotowa *et al*. (2013); Brozynska *et al*. (2014)]. Of these, Taxon A has plant and seed morphology similar to that of *O. rufipogon* and Taxon B appears to be similar to the annual *O. meridionalis*. Here, we report draft genomes for these two Australian wild rice taxa: Taxon A and Taxon B, which are likely to be novel and different species. The two taxa were sequenced and assembled by integration of two distinct next‐generation sequencing (NGS) data, namely Illumina and Pacific Biosciences. The draft nuclear genome sequences of 384.8 Mb (Taxon A) and 354.9 Mb (Taxon B) were placed on 12 pseudochromosomes based on available rice reference sequences. Taken together, this study creates a new genomic platform for investigating the gene pool and agriculturally important traits potentially present in Australian taxa.

**Figure 2:**
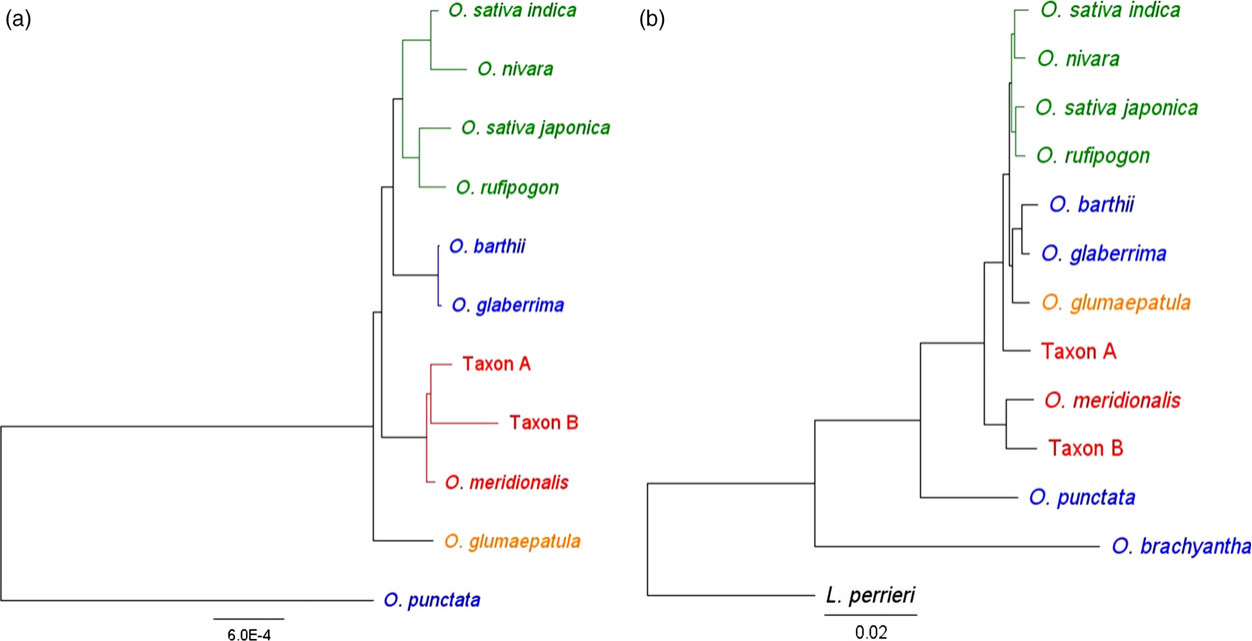
Phylogenetic relationships between A genome rice species; (a) tree topology based upon analysis of supermatrix of 4643 nuclear genes; (b) tree topology based on whole chloroplast genome sequences. Figure adapted and modified from Wambugu *et al*. (2015). Taxa marked in green represent Asian rice species, in blue: African, in orange: South American and in red: Australian. *L. perrieri* and *O. punctata* were used as outgroups in nuclear and chloroplast studies, respectively.

The Australian wild rices have been isolated from the impact of gene transfer from domesticated rice that may complicate interpretation of the genetics of wild rice populations in Asia where rice has been cultivated on a large scale for thousands of years. An understanding of genetic relationships and diversity between and within these Asian and Australian populations will guide the effective use of wild genetic resources for global rice improvement. The phylogenetic relationships between all of the A genome taxa have recently been estimated using whole chloroplast genome sequences (Brozynska *et al*., 2014; Wambugu *et al*., 2013). In this phylogeny, the Australian A genome taxa form a distinct clade, which is a sister to the Asian domesticated rice clade (Figure 2a). We now report a phylogenetic analysis of the corresponding nuclear genomes.

## Results

### Genome sequencing

The statistics of sequencing reads obtained in this study are shown in Table S1. Total data produced by the Illumina platform were 47.1 Gb and 41.4 Gb for Taxon A and Taxon B, respectively. The data generated on the PacBio instrument were long reads with an average length of 7693 bp and 8140 bp for Taxon A and Taxon B, respectively, with 14.8 Gb and 15.0 Gb of overall data for those taxa. The minimum and maximum read lengths for Taxon A were 50 bp and 49 742 bp, respectively, and for Taxon B: 50 bp and 50 242 bp.

The genome sizes were estimated *in silico* to be about 390 Mb and 370 Mb for Taxon A and Taxon B, in turn. These estimates were similar to other A genome rice species which fall between 341 and 413 Mb in size (Zhang *et al*., 2014). Considering the estimated genome sizes, we also assessed the genome coverage of each of the data sets (Table S2). Furthermore, we used these estimations in evaluating completeness of genome assemblies.

### Genome assemblies and evaluation

A total of 384.8 Mb (PacBio assembly) and 382.7 Mb (hybrid assembly) of the Taxon A, and 354.9 Mb (PacBio) and 446.4 Mb (hybrid) of the Taxon B genome sequences were assembled (Table 1). PacBio assemblies slightly outperformed hybrid assemblies in terms of standard assembly metrics, that is lower number of scaffolds, longest contig size, higher N50 and mean scaffold size. Both Taxon A and Taxon B assemblies exhibited high total lengths, as percentage of known genome sizes, with an unexpected high length of the Taxon B hybrid assembly that accounted for around 120% of estimated genome size (370 Mb). The high percentage of estimated genome size for Taxon B hybrid assembly may be due to the heterozygous and repetitive nature of this taxon's genome or to a nonprecise genome size estimation. Predicted heterozygous sites measured as the rate of variant branches caused by allelic differences in a de Bruijn graph, (1 in 400) and repeat content (1 in 300) rates were slightly higher for Taxon B compared to Taxon A (1 in 800 and 1 in 400, respectively; data not shown). Those traits might impact the assembly quality and completeness especially using short‐read data (Illumina) resulting in a more fragmented assembly with a higher number of repeated contigs.

**Table 1:** Taxon A and Taxon B hybrid and PacBio assembly statistics. The metrics were calculated for scaffolds and contigs for hybrid assembly and for scaffolds only for PacBio assembly

	Taxon A		Taxon B	
Assembly	Hybrid	PacBio‐only	Hybrid	PacBio‐only
Assembler	Sparse Assembler + DBG2OLC	Celera Assembler	Sparse Assembler + DBG2OLC	Celera Assembler
Scaffolds				
Number of scaffolds	3359	2585	4718	3252
Total size of scaffolds	382 655 312	384 759 810	446 369 637	354 906 376
Total scaffold length as percentage of known genome size	98.1	98.7	120.6	95.9
Longest scaffold	1 305 248	1 692 155	2 079 733	3 232 522
Shortest scaffold	2297	9523	2425	12 563
Mean scaffold size	113 919	148 843	94 610	109 135
Median scaffold size	61 996	97 803	54 787	61 207
N50 scaffold length	217 336	219 409	163 003	159 640
Contigs				
Number of contigs	3425	–	4808	–
Total size of contigs	382 644 322	–	446 351 110	–
Longest contig	1 158 569	–	1 449 836	–
Shortest contig	1139	–	790	–
Mean contig size	111 721	–	92 835	–
Median contig size	61 459	–	54 495	–
N50 contig length	211 599	–	159 759	–

The number of scaffolds obtained as a result of the assemblies was between 2585 and 3252 for Taxon A and Taxon B, respectively, for PacBio assemblies and between 3359 and 4718 for Taxon A and Taxon B, respectively, for hybrid assemblies. The number of scaffolds was slightly higher for hybrid assemblies for both taxa. Higher number of scaffolds in Taxon B assemblies might also be a result of the more heterozygous and repetitive nature of this taxon in comparison with Taxon A.

Both of the core gene presence evaluation methods, CEGMA and BUSCO, indicated PacBio assemblies to be more complete than hybrid assemblies for both taxa (Table S3 and Table S4). Normalized values ranged from 94.8% to 99.1% of completeness using CEGMA and from 89% to 98% using BUSCO. Neither wild rice assembly was found to have a higher number of mapped genes than the Nipponbare reference.

A high fraction of the Nipponbare reference genome was aligned to Taxon A assemblies, 70.7% and 71.5% to hybrid and PacBio, respectively (Table S5). However, a significantly lower percentage was aligned to Taxon B assemblies, 42.4% and 37.3% to hybrid and PacBio, respectively. These values were not high enough to use the Nipponbare genome as a reference for Taxon B in orienting and ordering contigs into chromosome pseudomolecules using Genome Puzzle Master [(GPM; Zhang *et al*. (2016)]. Based on the previous finding that Taxon B shares numerous molecular markers with *O. meridionalis* (Sotowa *et al*., 2013) and that they descended from a common ancestor (this study), this genome was evaluated as well. QUAST results showed higher *O. meridionalis* genome fraction aligning to Taxon B assemblies than Nipponbare genome, 62.4% and 56.3% to hybrid and PacBio, respectively. These values were satisfactory allowing the use of *O. meridionalis* instead of Nipponbare sequences as a guide in GPM for Taxon B assembly.

### Rice pseudomolecules

After the preliminary evaluation of the assemblies (basic assembly statistics, core gene presence and alignment to the reference genomes), the PacBio‐only scaffolds were chosen for further analysis and investigation. Ordering and orientation of the contigs with GPM resulted in 12 pseudochromosomes for both taxa and 386 unordered contigs for Taxon A and 1080 for Taxon B (Table S6). 94.9% and 83.1% of the assemblies’ length were anchored and oriented to chromosomes for Taxon A and Taxon B, respectively. Alignment of wild rice pseudomolecules to their reference genomes revealed better coverage and less ambiguity between Taxon A and *O. sativa japonica* than between Taxon B and *O. meridionalis* genome (Figure S1).

### Genome annotation

Repetitive elements, RNAs and protein coding genes were annotated in Taxon A and Taxon B draft genomes. The sequences subjected to the annotation were the 12 pseudomolecules and remaining unordered contigs for each of the taxa.

Total repeats found in Australian wild rices made up 36.5% and 46.4% of the Taxon A and Taxon B genomes, respectively (Table S7 and Table S8). The most abundant class of transposable elements found were retrotransposons from the Gypsy superfamily. These represented 57.8% and 39.7% of all repeats described in Taxon A and Taxon B, respectively, followed by the Copia superfamily in Taxon A (8.3%) and Mutator in Taxon B (9.3%). The classes and fractions of other repetitive elements were similar in both taxa; however, the numbers and lengths were significantly higher in the Taxon B genome.

Noncoding RNAs annotated in the wild rice genomes included tRNA, miRNA, snoRNA, sRNA, rRNA and other (Table S9). Overall, RNAs consisted of approximately 0.25% of both genomes which corresponded to length of 960 496 bp in Taxon A and 883 779 bp in Taxon B. 675 and 558 tRNAs models were predicted by tRNAscan in Taxon A and Taxon B, respectively, whereas 629 and 581 tRNAs models were predicted by Infernal, respectively. Additional models predicted by Infernal but not by tRNAscan were added to the final annotations resulting in 677 and 615 tRNAs for Taxon A and Taxon B, respectively, of the combined length of 50 687 and 46 301 bp.

The number of gene models annotated in wild rice genomes is listed in Table S10. Slightly more genes were found in the Taxon A genome which was probably associated with the longer total assembly of this taxon (384.8 Mb as opposed to 354.9 Mb of Taxon B). In comparison with other wild rice species (I‐OMAP, unpublished), these taxa showed a considerably lower number of genes. In the previous study, the lowest number of annotated loci was found in *O. brachyantha* (24 208), which also carries the smallest genome described so far in the genus *Oryza* (261 Mb). Similar numbers of InterPro protein domains, KEGG pathways or GO terms were found in the two genomes (Table S10). Overall, just over 61% of annotated models had matches in the InterPro database, about 9% in KEGG and around 40% in GO.

### Phylogenetic analysis

Sequences of 4643 genes were extracted from nuclear genome sequences generated by whole genome sequencing (I‐OMAP, unpublished; Table S11). The alignment of these 4643 gene sequences had a total length of 6 272 851 bp. The sequence similarity between the *Oryza* species was very high (Table 2) ranging from 86.6% (between *O. meridionalis and O. brachyantha*) to up to 98.4% (between *O. rufipogon* and *O. sativa* ssp. *japonica*). Of the bases that were subjected to the maximum parsimony (MP) analysis, 5 229 706 were constant, 741 498 were variable and parsimony uninformative, and 301 647 were parsimony informative. Both phylogenetic inference methods used in this study, maximum parsimony (MP) and Bayesian inference (BI), recovered the same optimal tree topology (Figure 2b) with the following values for the MP tree: length = 1 264 556 steps, consistency index CI = 0.90, retention index RI = 0.74, CI excluding uninformative characters = 0.72. The nodes on this topology were all strongly supported with MP bootstrap values of 100% and the posterior probabilities of all nodes in the BI equal to 1.

**Table 2:**
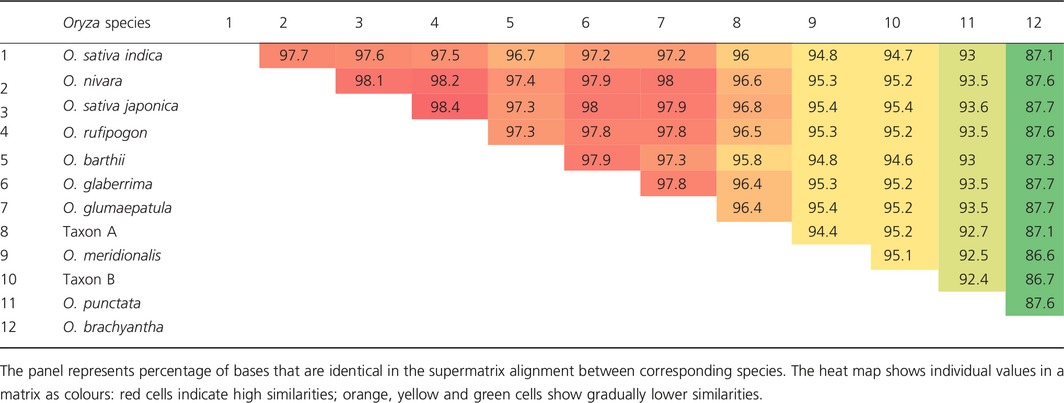
Sequence similarities between rice taxa in the supermatrix used for phylogenetic inference

Our results showed that *O. meridionalis* and Taxon B in Australia are sister to all other A genome species including the Australian Taxon A (Figure 2b). Taxon A is in turn a sister to the clade that includes the Asian and African domesticated species. African domesticated rice, *O. glaberrima*, and its wild progenitor, *O. barthii*, together with *O. glumaepatula* from South America are a clade distinct from the Asian species (Wambugu *et al*., 2013). *Indica* and *japonica* rice are represented by two well‐resolved clades. *Japonica* and *O. rufipogon* show a close relationship which is consistent with the long‐accepted view that *O. rufipogon* is the progenitor of *japonica* (Wei *et al*., 2012) while *indica* rice was found in a clade with the Asian annual *O. nivara*. Recent SNP analysis of genomic regions under selection suggests the independent domestication of *indica* rice from wild rice in an area from southern Indochina to the Brahmaputra valley (Civáň *et al*., 2015).

The relationship of the Australian A genome populations was greatly clarified by this study. *O. meridionalis* and the morphologically similar perennial populations (Taxon B) are sister to all other A genome species. The Australian wild populations with morphology similar to *O. rufipogon* (Taxon A) were found to be sister to the clades including all other A genome species. The Australian (Taxon A) population has a large anther like that of *O. rufipogon* in Asia but is morphologically distinct from the other Australian A genome species and domesticated rice with small anthers. Taxon A and Taxon B can be most readily distinguished in the field by the open panicles of Taxon A and closed panicles of Taxon B (Figure 1). Taxon B generally has longer awns, but the ranges of awn length for the two taxa overlap. The presence of these diverse A genome taxa makes northern Australia a key centre of diversity for rice and indicates the need for more collections from this poorly explored area and the need to ensure in situ conservation of these resources.

The nuclear phylogenies presented here showed a different relationship to those deduced from the chloroplast genomes suggesting different evolutionary histories for the maternally inherited plastid genetic material and the nuclear genome (Figure 2). This difference in evolutionary path for these genomes is commonly observed in recently diverged plant taxa (Tsitrone *et al*., 2003).

**Figure 3:**
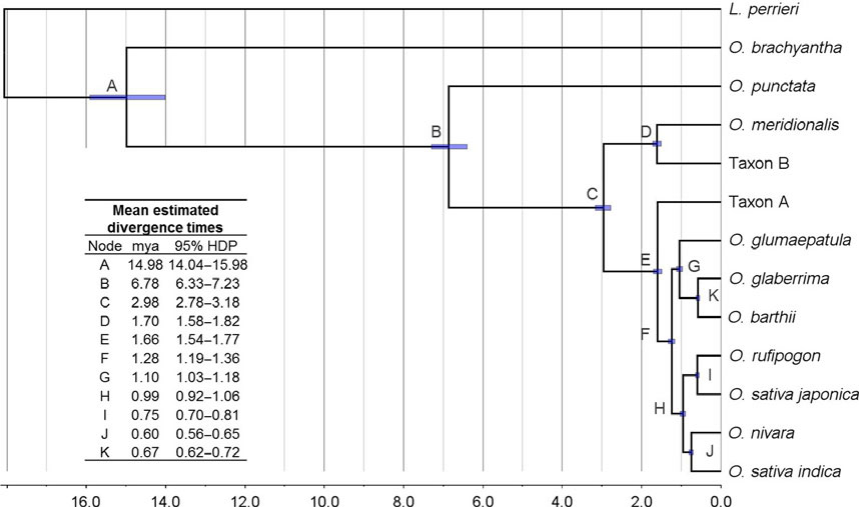
Molecular clock analyses for A genome rice evolution. The most frequent tree topology retrieved in analyses of alignments of separate chromosomes, inferred for 8 out of 12 chromosomes. Scale axis represents age in million years (mya). Node bars display 95% highest posterior density (HDP) interval.

Analysis of the timing of the evolutionary events in this study (Figure 3) agrees well with that reported in analysis of the *Oryza* genomes (I‐OMAP, unpublished) despite the use of a different method of analysis. The average rate of evolution was estimated to be 3.53E‐3 ± 1.85E‐6 (Table S12) and the root age (divergence between *O. brachyantha* and *O. punctata*) to be 14.98 ± 0.97 mya (Figure 3). Given this root age, the A genome group diverged in the last 3 million years and the divergence of the *japonica* and *indica* clades dated at about 990 000 years ago (Figure 3). The chloroplast genomes appear to have diverged more recently (Wambugu *et al*., 2015) possibly due to some degree to the sharing of maternal genomes across this group.

**Figure 4:**
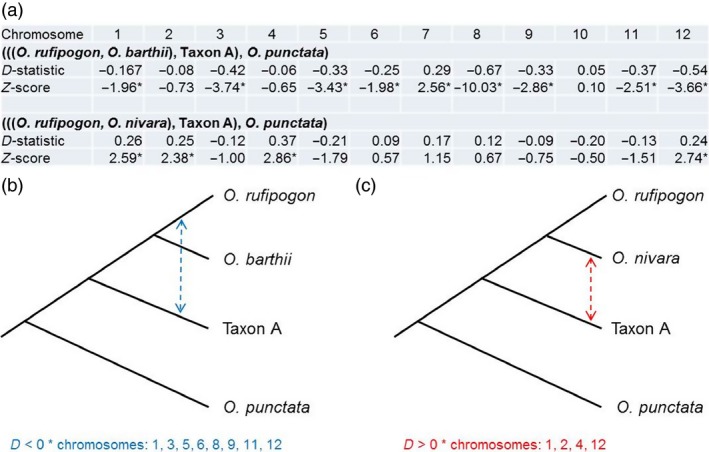
Results of four‐taxon test for Taxon A and selected *Oryza* species; (a) D‐statistics and *Z*‐scores calculated for two sets of selected species per chromosome: (((*O. rufipogon*,* O. barthii*), Taxon A), *O. punctata*) and (((*O. rufipogon*,* O. nivara*), Taxon A), *O. punctata*). *Z*‐scores marked with asterisk (*) indicate statistically significant values; (b) four‐taxon tree used in the first test. Bidirectional arrow shows inferred introgression for chromosomes with significant D‐statistics (listed below the tree); (c) four‐taxon tree used in the second test. Bidirectional arrows show inferred introgression for chromosomes with significant D‐statistics (listed below the tree).

Analysis of the phylogenetic relationship for each chromosome separately revealed some discordant results for chromosomes 5, 7, 10 and 11 (Figure S2, Table S13) that is likely due to introgression rather than incomplete lineage sorting. To test for potential recombination events between Australian Taxon A and other *Oryza* species, we performed a four‐taxon test, also known as the D‐statistic (Durand *et al*., 2011; Green *et al*., 2010) separately for each chromosome. This test screens the aligned data for two biallelic mutation patterns: ABBA and BABA. The first species set we used was ((*O. rufipogon*,* O. barthii*) Taxon A, *O. punctata*) where *O. punctata* was used as the outgroup. In this analysis, a negative D‐statistic value would suggest introgression between Taxon A and the Asian species, whereas a positive value would mean an introgression between Taxon A and the African species (*O. barthii*). In this s*et, a*ll but two chromosomes showed negative D‐statistic values, indicating that introgression occurred between Australian Taxon A and Asian *O. rufipogon* (Figure 4, Table S14). Eight of these statistics were significant (chromosomes 1, 3, 5, 6, 8, 9, 11 and 12). Among chromosomes with significant results was chromosome 5 for which Taxon A was found to be closer on the phylogenetic tree to the Asian clade (Figure S2) than it was on the consensus tree (Figure 2b). The D‐statistic calculated for chromosome 7 was positive and statistically significant, thereby providing evidence for introgression between Taxon A and *O. barthii*.

To investigate the relationship between Australian and Asian species, we used the set ((*O. rufipogon*,* O. nivara*), Taxon A, *O. punctata*). For this set, a negative D‐statistic value would suggest introgression between Taxon A and the *japonica*/*O. rufipogon* clade, whereas a positive value would suggest introgression between Taxon A and the *indica*/*O. nivara* clade. Four chromosomes (1, 2, 4 and 12) returned significant positive values, whereas no negative value was meaningful. Introgression between the Asian and Australian populations was suggested with evidence for greater introgression between Taxon A and the *indica*/*O. nivara* clade than between Taxon A and the *japonica*/*O. rufipogon* clade.

## Discussion

The discovery of two novel Australian wild rice taxa expands the understanding of the genetic diversity within the genus *Oryza*. The two draft genomes generated in this study produce an excellent platform for exploring the potential of Australian wild rices. These genomic resources will complement the already extensive study of numerous wild and cultivated species within *Oryza* (I‐OMAP, unpublished) providing unique data for comparative genomics, evolutionary studies of the entire genus and widening the species pool for rice improvement.

The sequencing and assembly performed in this study show the particular challenges of generating genome assemblies for wild heterozygous plants. It also shows that the replacement of short‐read data (Illumina) with long‐read data (PacBio) can improve the overall completeness of a draft genome sequence. The results suggest that the best representation of the Taxon A and Taxon B genomes was obtained using the PacBio‐only data. As a consequence, the PacBio‐only assemblies for both taxa have been selected for further analysis. PacBio‐only assembly improved almost all metrics, such as number of scaffolds, total size, the longest and the shortest scaffolds, mean and median lengths as well as completeness measured as presence or absence of orthologous genes.

The evolution of rice has been the subject of ongoing debate in particular regarding whether there was a single or multiple domestication in Asia (Bouchenak‐Khelladi *et al*., 2010; Kellogg, 2009; Vaughan *et al*., 2008). The distinctness of the *indica* and *japonica* genomes suggests separate origins for most of each genome (Wei *et al*., 2012). However, the presence of many shared domestication‐related alleles has led to suggestions that some level of introgression between the two genomes has also been a feature of their domestication history (Civáň *et al*., 2015; Fuller *et al*., 2010; Huang *et al*., 2012a,b; Molina *et al*., 2011). Geographic separation may have allowed early populations to diverge resulting in distinct *O. rufipogon*‐like populations in Asia and Australia. Chloroplast transfer or capture is common between recently diverged plant taxa which may explain the distinct Asian and Australian chloroplast genomes in the wild populations descended from the taxa that were domesticated in Asia.

Wild rice populations are a key genetic resource for rice improvement (Anacleto *et al*., 2015). The Australian populations may provide an especially useful resource for evaluation of rice domestication due to their isolation from significant impact of gene flow from domesticated rice. An improved understanding of the geographic variation in A genome wild rice species provided by genome sequencing should guide the search for useful alleles in wild populations (Krishnan *et al*., 2014). The taxonomy of the A genome wild rice species in Australia and Asia needs to be re‐evaluated in the light of the molecular data now available to determine whether distinct wild taxa need to be recognized. Movement of flora between Sahul (New Guinea and Australia) and Sunda (Malay Peninsula, Sumatra, Borneo, Java) in both directions may be an important part of the evolutionary history of the A genome *Oryza* species (Crayn *et al*., 2015; Prasad *et al*., 2011; Tang *et al*., 2010). The current diversity of A genome *Oryza* in northern Australia suggests the possibility of an Australian and/or South‐East Asian origin for the A genome clade, but further historical biogeographical analyses based on more extensive data sets are required to evaluate this hypothesis.

## Experimental procedures

### Plant material

The wild rice plants used in the study came from perennial wild rice populations in North Queensland, Australia. The first individual, referred to here as Taxon A, was collected from Abattoir Swamp Environmental Park near Julatten and was described by Sotowa *et al*. (2013) as *Oryza rufipogon*‐like taxon (r‐type) collected from Jpn1 site. The second individual, referred to here as Taxon B, was collected from a small wetland beside the Peninsula Developmental Road and in Sotowa *et al*. (2013) was called *Oryza meridionalis*‐like (m‐type) taxon collected from Jpn2 site. Specimens of these wild rice populations were collected from their natural habitats and are now kept and maintained in glasshouse conditions at The University of Queensland in Brisbane, Australia.

### DNA extraction and sequencing

DNA from leaf tissue of Taxon A and Taxon B individuals was extracted using a modification of the CTAB method (Furtado, 2014) and subsequently subjected to whole genome shotgun sequencing. Next‐generation sequencing platforms used were Illumina HiSeq2000 (Illumina, San Diego, CA) and Pacific Biosciences RSII with P6‐C4 chemistry (PacBio, Menlo Park, CA). The data generated on Illumina instrument were 101‐bp reads with an average library insert of 550 bp (paired end reads, PE), 3000 bp (mate pair reads, 3 Kb MP) and 5000 bp (mate pair reads, 5 Kb MP). Samples for paired end sequencing were generated using TruSeq DNA PCR‐free library preparation kit, whereas mate pair libraries were prepared using the Nextera Mate Pair protocol. Illumina sequencing was performed by Macrogen (Seoul, Korea) and PacBio sequencing by The University of Queensland Diamantina Institute (Brisbane, Australia). The SMRTbell template libraries were prepared following the standard protocol for long‐insert libraries according to the manufacturer's instructions (PacBio) with an insert size of 20 kbp. 20 SMRT Cells per taxon were sequenced resulting in approximately 40‐fold genome coverage for each sample.

### Data processing and genome assembly

Raw reads from both platforms were assessed using FastQC (www.bioinformatics.babraham.ac.uk/projects/fastqc), a tool for evaluating the quality of sequencing reads in FASTQ files. Illumina reads were additionally used to estimate the genome size of the taxa. A preqc module (Simpson, 2014) from the SGA de novo genome assembler package (Simpson and Durbin, 2012) was used for this estimation. This utility also enabled an estimation of heterozygosity and repeat content in the genome.

Taxon A and Taxon B genomes were assembled *de novo* using two strategies. The first one utilized Illumina and PacBio sequencing reads together (hybrid assembly) and the second one – PacBio data only (PacBio‐only assembly). The software used to accomplish the hybrid assembly was DBG2OLC package (Ye *et al*., 2016). This assembly included Illumina PE raw reads and raw PacBio reads. The first step within the analysis involved two rounds of Illumina read error correction and subsequent assembly with SparseAssembler [beta version; Ye *et al*. (2012)]. The coverage threshold for both an error and for a correct sparse *k*‐mer candidate in the correction process was set to 5. In the first round, the *k*‐mer length was set to 15 and reads were trimmed at the ends, whereas in the second one, the *k*‐mer length used was 31 and the trimming option was disabled. *K*‐mer size in the assembly step was 31. The next step was ‘overlap and layout’ with the output contigs from the first step and PacBio reads with the following parameters: *k*‐mer = 17, k‐mer coverage threshold = 2, adaptive threshold = 0.001, minimum overlap = 20 and removal of chimeric reads in the data set. The last step called the consensus contigs from output files from two previous steps and raw PacBio reads. In PacBio‐only assembly, first, the raw reads from the PacBio platform were corrected using the PBcR pipeline (Berlin *et al*., 2015) with the self‐correction feature enabled and the minimum length of PacBio fragment to keep set to 500. The assembly was done using The Celera Assembler (CA) version 8.3rc2 (Myers *et al*., 2000) leaving the parameters as default. The primary contigs were filtered in order to keep only the unique contigs.

### Evaluation of genome assemblies

First, each of the assemblies was assessed using Assembly Stats (assemblathon tool) from the Assemblathon project (Earl *et al*., 2011) accessed through the iPlant Collaborative platform [iPlant; Goff *et al*. (2011)]. The expected genome sizes were set based on the estimations from this study. Second, core gene presence was assessed in the assemblies. This was done using both CEGMA [Core Eukaryotic Genes Mapping Approach; Parra *et al*. (2007)] and BUSCO [Benchmarking Universal Single‐Copy Orthologs; Simao *et al*. (2015)], keeping the default cut‐offs for genes. CEGMA uses a set of 248 CEGs (core eukaryotic genes) which are very highly conserved in eukaryotes and are present in low copy number (Parra *et al*., 2009). BUSCO also uses a set of universal single‐copy orthologs and provides a set to evaluate plant genomes in particular with a set of 956 plant orthologs. Additionally, both evaluation tools were run for the rice reference sequence of *O. sativa japonica* and the values for completeness of this high quality rice genome were used for normalization.

The assemblies were also aligned to rice reference genomes using QUAST [Quality Assessment Tool for Genome Assemblies; Gurevich *et al*. (2013)] with the default parameters and the minimum alignment length of 1000 bp. The genome of *Oryza sativa* ssp. *japonica* var. Nipponbare [IRGSP_MSU.v7; Kawahara *et al*. (2013)] and *O. meridionalis* (GenBank assembly accession: GCA_000338895.2) was used as the reference sequences in this study.

### Rice pseudomolecules

Assembled PacBio contigs were assigned to chromosome pseudomolecules using Genome Puzzle Master [GPM; Zhang *et al*. (2016)]. Rice reference genome sequences were used to guide the process: *Oryza sativa* ssp. *japonica* var. Nipponbare genome was used for Taxon A and *O. meridionalis* was used for Taxon B. The twelve pseudomolecules and unanchored contigs were then annotated for genes and other features.

### Genome annotation

Protein coding genes were annotated using the MAKER‐P v.2.3 annotation pipeline (Campbell *et al*., 2014). Within the pipeline, the repeat elements were masked using the RepeatMasker (www.repeatmasker.org, v. 3.3.0). Due to the lack of expression data for these specific taxa, expression evidence included available expressed tags (ESTs) and full‐length cDNA from other *Oryza* taxa. These CDS and their corresponding protein sequences (used as the protein homology evidence) consisted of annotated genes models of *O. sativa* ssp. *japonica* var. Nipponbare RefSeq, *O*. *glaberrima* (Wang *et al*., 2014) and *Brachypodium distachyon* (The International Brachypodium Initiative 2010). ESTs comprised of *O. sativa* ssp. *japonica* var. Nipponbare (for Taxon A annotation) and *O. meridionalis* (for Taxon B annotation) transcripts generated by The International *Oryza* Map Alignment Project (I‐OMAP, unpublished) and clustered at 95% similarity. The *ab initio* gene predictors run within MAKER‐P were SNAP (Korf, 2004) using O. sativa.hmm parameter and AUGUSTUS 3.1 (Stanke and Waack, 2003) with rice as the gene prediction species model. Resulting gene models were filtered removing noncomplete models, that is without valid start and/or stop codons and with internal terminator codons, followed by removing transposable elements (TE) based on the specific rice‐ and Australian taxa‐related libraries used at the genome masking step. Removed TE consisted of hits above the e‐value threshold of 1e‐5, with more than 40% of query coverage and longer than 100 nt. The gene models predicted by MAKER‐P were functionally analysed using InterProScan version 5.16.55 (Jones *et al*., 2014) including annotation with Gene Ontology (GO) and biological pathway information. The InterProScan results were further parsed for additional functional evidence (GO terms and KEGG pathway) using interproscanParser script available at iPlant.

The repeat annotation was obtained by merging the output of RepeatMasker and Blaster, a component of the REPET package (Flutre *et al*., 2011), using nucleotide libraries (PReDa and RepeatExplorer) from RiTE‐db (Copetti *et al*., 2015) and an in‐house curated collection of transposable element (TE) proteins. Additionally, for each of the two species, a custom repeat library was developed with RepeatExplorer and curated as described previously (Copetti *et al*., 2015) using short‐read data (Illumina sequencing reads). Infernal (Nawrocki and Eddy, 2013) was adopted to identify noncoding RNAs (ncRNAs) using the Rfam library Rfam.cm.1_1. Hits above the e‐value threshold of 1e‐5 were filtered, as well as results with scores lower than the family‐specific gathering threshold. When loci on both strands were predicted, only the hit with the highest score was kept. Transfer RNAs were also predicted using tRNAscan‐SE v. 1.23 (Schattner *et al*., 2005) with default parameters.

### Phylogenetic analysis

Phylogenetic analysis was undertaken using data from twelve fully sequenced diploid *Oryza* genomes including two taxa investigated in this study (Taxon A and Taxon B), and ten other species downloaded from GenBank (Table S11). Eleven of these species were A genome‐type rice relatives. Moreover, we used *O. punctata*, which belongs to BB genome group, and *O. brachyantha*, which has FF genome type. *Leersia perrieri* was used as the outgroup species.

From these diploid genomes, a set of putatively single‐copy orthologs was selected by blasting (BLASTn) the initial collection of 6,015 genes (representing the set of genes that could be identified in all *Oryza* taxa) used in a previous study (I‐OMAP, unpublished) against the Australian *Oryza* genomes (Taxon A and Taxon B) applying the following thresholds: e‐value of 1e‐5, 40% of query coverage and 100 nt of hit length. The initial set of clusters of single‐copy orthologous loci was identified by BLAST‐Overlap‐Synteny (BOS) filtering using a protocol described by Zwickl *et al*. (2014). A final subset of 4,643 genes sequences present in all *Oryza* assemblies was extracted from the genomes and used for further investigation. Nucleotide sequences of the 4,643 genes selected from each genome were separately aligned using CLUSTALW multiple sequence alignment program (Thompson *et al*., 1994) with default parameters. Then, single gene alignments were concatenated to create a supermatrix of 6 272 851 base pairs, which was used in the following phylogenetic inference.

Phylogenetic tree reconstruction was conducted using maximum parsimony (MP) and Bayesian inference (BI) methods. MP was performed using PAUP* 4.0 software (Swofford, 2003). The following tree search settings were enabled in MP reconstruction: heuristic search with tree bisection–reconnection branch swapping and 200 random addition sequence replications. The group support was assessed using 2000 bootstrap pseudoreplications. Alignment gaps were treated as missing data. All characters were treated as unordered and weighted equally.

For the model‐based approach (BI), jModelTest2 software (Darriba *et al*., 2012) was used to determine the model of nucleotide substitution that best fits the data based on the Akaike information criterion. The Bayesian analyses used the general time reversible model with gamma‐shaped among‐site rate variation with an estimated proportion of invariable sites (GTR+I+G; p‐inv = 0.3730, four gamma categories and gamma shape = 0.8890). The BI analysis was performed using MrBayes version 3.2 (Ronquist *et al*., 2012). The branch length prior was set to exponential with parameter 10.0. Two independent and simultaneous analyses starting from distinct random trees were performed. Three heated (heating coefficient = 0.2) and one cold Monte Carlo Markov chains (MCMC) were run for 1 × 10^6^ generations, with a tree sampled every 200 generations. The first 10% of trees were discarded as burn‐in and a 50% majority rule consensus tree was constructed and rooted using the outgroup method.

### Divergence time estimates

Divergence times were estimated using the Bayesian evolutionary method implemented in the software package BEAST 2 version 2.3.1 (Bouckaert *et al*., 2014). A secondary clock calibration was used based on the estimated divergence time for the *Oryza* crown group (*O. brachyantha*–*O. punctata*) of 15 ± 0.5 mya (I‐OMAP, unpublished). The 4643 genes used were divided according to the chromosome they were found on and aligned using CLUSTALW. The number of genes and the alignment length is shown in Table S15. The best fit evolutionary model, determined by jModelTest2, was the general time reversible model (GTR+I+G) for each of the alignments. Evolutionary rates were modelled under a strict molecular clock and speciation was modelled employing the Yule model. Posterior probabilities were estimated using MCMC algorithm with chain length 5 000 000 and a tree sampled every 1000th generation. The first 10% of sampled trees was discarded as burn‐in. The output from BEAST 2 was analysed in Tracer version 1.63 (www.beast.bio.ed.ac.uk/Tracer). The best supported tree with the highest product of the posterior probability of all its nodes (maximum clade credibility tree) and the mean heights of each node was summarized using TreeAnnotator distributed in the BEAST 2 package. The final tree estimates were visualized in FigTree version 1.4.2 (www.tree.bio.ed.ac.uk/software/figtree).

### Genetic introgression

To test for genetic introgression between Australian and other *Oryza* species, we used the software package HYBRIDCHECK (Ward and van Oosterhout, 2016). We performed two tests and in each run four aligned sequences were analysed. In the first one, we tested for introgression between Taxon A and either *O. rufipogon* or *O. barthii,* and in the second, we tested for introgression between Taxon A and either *O. rufipogon* or *O. nivara*. Block jackknife was used to calculate the statistic with the block size of 20 000 as well as *Z*‐score to measure the statistical significance.

## Supporting information


**Figure S1** Dot plot of wild rice pseudomolecules with reference genomes chromosomes; (a) Taxon A and its reference, *O. sativa* ssp. *japonica* var. Nipponbare; (b) Taxon B and its reference, *O. meridionalis*. Each square corresponds to one of the 12 rice chromosomes.

**Figure S2** Bayesian phylogenies for each chromosome of investigated *Oryza* species and the outgroup. Tree topologies for chromosomes 1, 2, 3, 4, 6, 8, 9 and 12 are identical. Tree topologies for chromosomes 5, 7, 10 and 11 are distinct and are highlighted in yellow. Scale axis represents age in million years (mya). Node bars display 95% Highest Posterior Density (HDP) interval.

**Table S1** Illumina and PacBio sequencing reads statistics for Taxon A and Taxon B.

**Table S2** Illumina and PacBio sequencing genome coverage for Taxon A and Taxon B calculated using the estimated genome sizes (390 Mb and 370 Mb for Taxon A and Taxon B, respectively).

**Table S3** Completeness of Taxon A and Taxon B assemblies evaluated by means of presence of CEGMA core genes.

**Table S4** Completeness of Taxon A and Taxon B assemblies evaluated by means of presence of BUSCO orthologous genes.

**Table S5** Unaligned and partially unaligned contig metrics of Australian wild rice taxa.

**Table S6** Length and GC content of Taxon A and Taxon B pseudomolecules and remaining contigs.

**Table S7** Repetitive elements annotated in Taxon A genome.

**Table S8** Repetitive elements annotated in Taxon B genome.

**Table S9** Non‐coding RNA annotation in Taxon A and Taxon B genomes.

**Table S10** Protein coding genes annotation in Taxon A and Taxon B genomes.

**Table S11** *Oryza* and the outgroup species used in the phylogenetic study.

**Table S12** The average rate of evolution estimates for *Oryza* species by chromosome.

**Table S13** Divergence times estimates for *Oryza* species by chromosome.

**Table S14** Global statistics calculated in four‐taxon test for selected *Oryza* species per chromosome.

**Table S15** Summary of data used in divergence time estimation.
