# Supplementary figures and images for: Sequencing of Australian wild rice genomes reveals ancestral relationships with domesticated rice
Source: Plant Biotechnol J. 2017 Jan 23;15(6):765–74. doi: 10.1111/pbi.12674 (PMC5425390; doi:10.1111/pbi.12674)

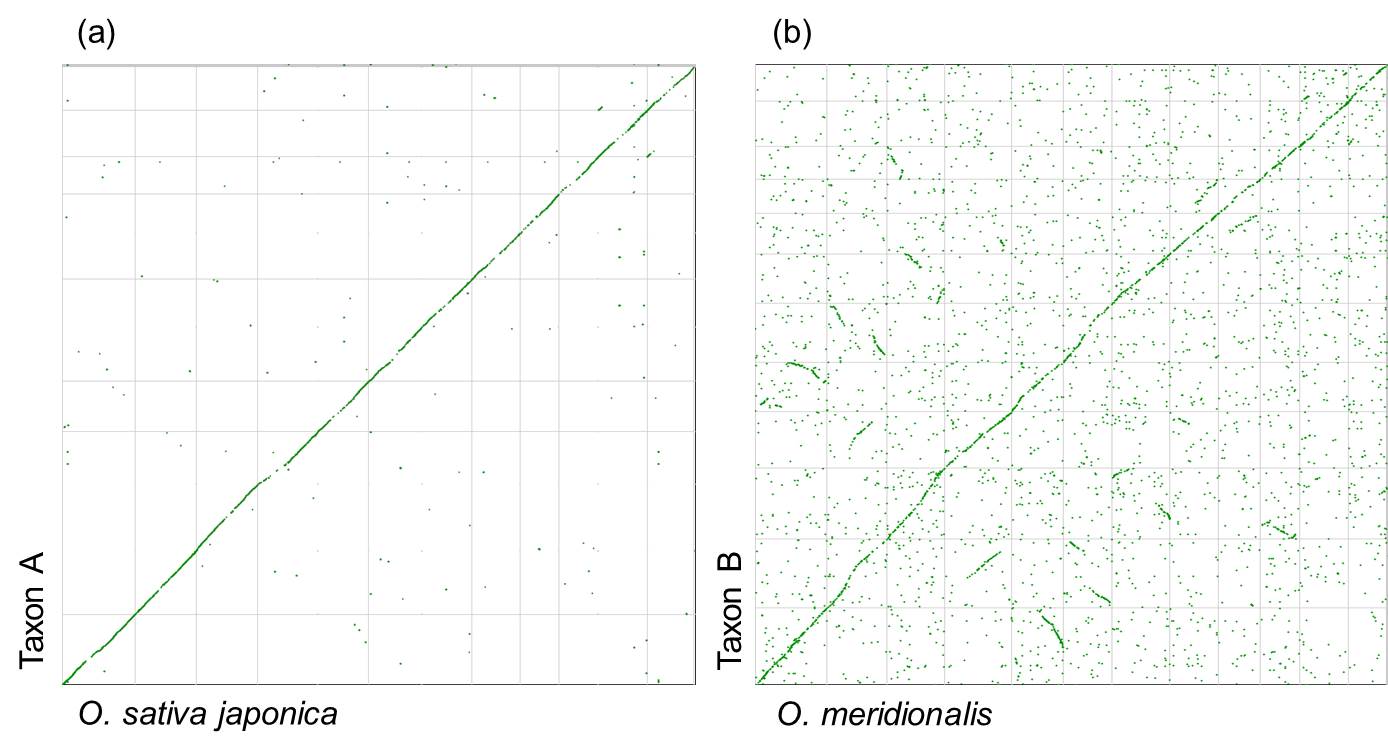

Supplement: Supplementary file 1 — Figure S1 Dot plot of wild rice pseudomolecules with reference genomes chromosomes; (a) Taxon A and its reference, O. sativa ssp. japonica var. Nipponbare; (b) Taxon B and its reference, O. meridionalis. Each square corresponds to one of the 12 rice chromosomes. [file PBI-15-765-s001.jpg]

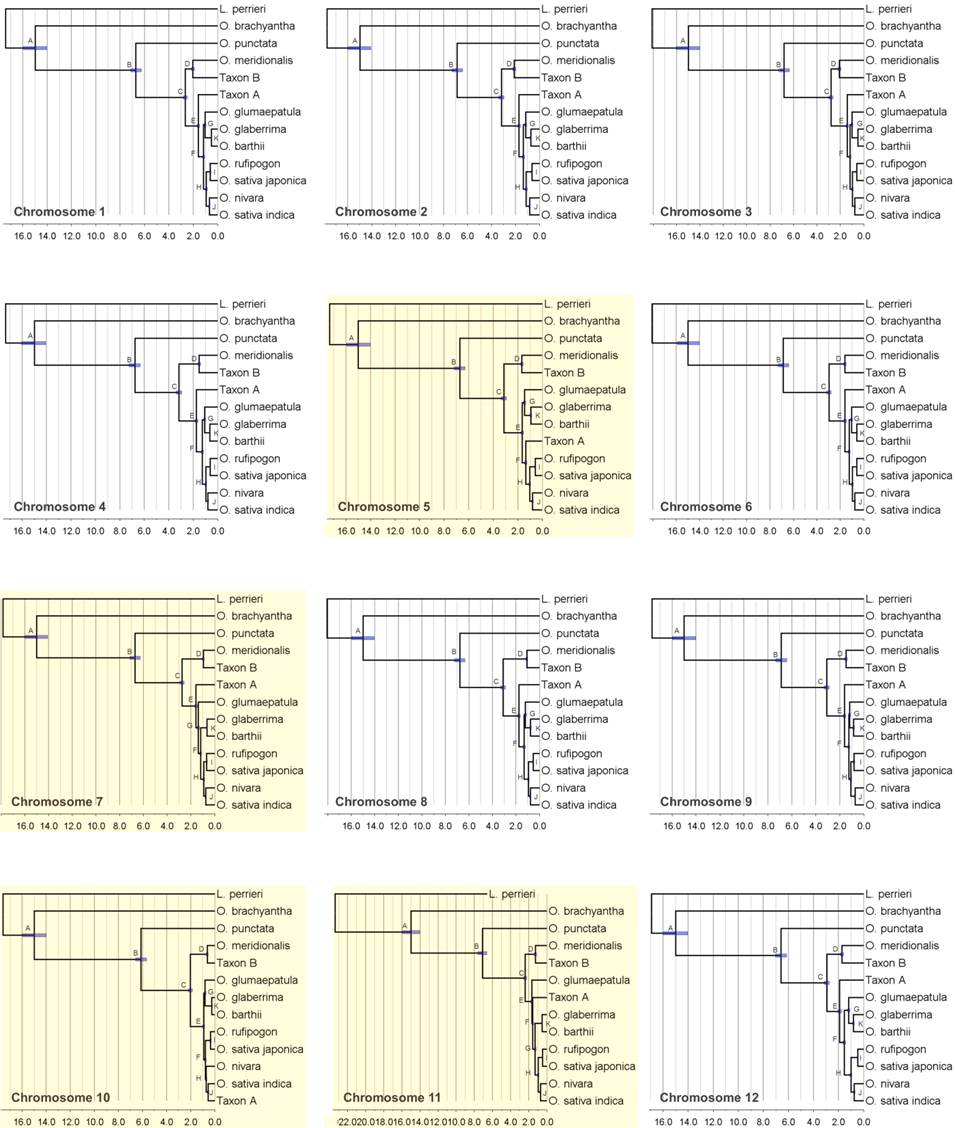

Supplement: Supplementary file 2 — Figure S2 Bayesian phylogenies for each chromosome of investigated Oryza species and the outgroup. Tree topologies for chromosomes 1, 2, 3, 4, 6, 8, 9 and 12 are identical. Tree topologies for chromosomes 5, 7, 10 and 11 are distinct and are highlighted in yellow. Scale axis represents age in million years (mya). Node bars display 95% Highest Posterior Density (HDP) interval. [file PBI-15-765-s015.jpg]
