# Supplementary material for: Sequencing of Australian wild rice genomes reveals ancestral relationships with domesticated rice
Source: Plant Biotechnol J. 2017 Jan 23;15(6):765–74. doi: 10.1111/pbi.12674 (PMC5425390; doi:10.1111/pbi.12674)
Supplement: Supplementary file 3 — Table S1 Illumina and PacBio sequencing reads statistics for Taxon A and Taxon B. [file PBI-15-765-s014.pdf]

Table S1 Illumina and PacBio sequencing reads statistics for Taxon A and Taxon B.

	# reads	# bases	Avg. length (bp)	Avg. GC (%)	Avg. ambiguous base (%)	Avg. quality (PHRED)	Quality distribution (PHRED)
**Illumina**							
Taxon A							
PE	200,095,430	20,209,638,430	101	43	0.11	35	10-40
3 Kb	155,539,872	15,709,527,072	101	44	0.04	35	2-40
5 Kb	110,916,270	11,202,543,270	101	44	0.04	35	2-40
Total	466,551,572	47,121,708,772	101	43	0.06	35	2-40
Taxon B							
PE	172,599,982	17,432,598,182	101	42	0.11	35	10-40
3 Kb	119,259,720	12,045,231,720	101	44	0.04	35	2-40
5 Kb	118,252,654	11,943,518,054	101	43	0.04	35	2-40
Total	410,112,356	41,421,347,956	101	43	0.06	35	2-40
**PacBio**							
Taxon A							
	1,928,732	14,781,976,594	7,664	43	NA	10	0-15
Taxon B							
	1,867,655	15,002,306,864	8,140	43	NA	10	0-15

PE – paired end reads, 3 Kb – mate pair reads with 3 Kb insert size, 5 Kb – mate pair reads with 5Kb insert size, NA – no ambiguous bases detected.
