# Supplementary material for: Sequencing of Australian wild rice genomes reveals ancestral relationships with domesticated rice
Source: Plant Biotechnol J. 2017 Jan 23;15(6):765–74. doi: 10.1111/pbi.12674 (PMC5425390; doi:10.1111/pbi.12674)
Supplement: Supplementary file 4 — Table S2 Illumina and PacBio sequencing genome coverage for Taxon A and Taxon B calculated using the estimated genome sizes (390 Mb and 370 Mb for Taxon A and Taxon B, respectively). [file PBI-15-765-s016.pdf]

Table S2 Illumina and PacBio sequencing genome coverage for Taxon A and Taxon B calculated using the estimated genome sizes (390 Mb and 370 Mb for Taxon A and Taxon B, respectively).

**Taxa**	**Genome coverage (x)**				
	**Illumina**				**PacBio**
	PE	3 Kb	5 Kb	Total	Total
Taxon A	51.8	40.3	28.7	120.8	37.9
Taxon B	47.1	32.6	32.3	112.0	40.6

PE – paired end reads, 3 Kb – mate pair reads with 3 Kb insert size, 5 Kb – mate pair reads with 5Kb insert size
