# Supplementary material for: Sequencing of Australian wild rice genomes reveals ancestral relationships with domesticated rice
Source: Plant Biotechnol J. 2017 Jan 23;15(6):765–74. doi: 10.1111/pbi.12674 (PMC5425390; doi:10.1111/pbi.12674)
Supplement: Supplementary file 5 — Table S3 Completeness of Taxon A and Taxon B assemblies evaluated by means of presence of CEGMA core genes. [file PBI-15-765-s017.pdf]

Table S3 Completeness of Taxon A and Taxon B assemblies evaluated by means of presence of CEGMA core genes.

Taxa	Assembly	Mapped CEGs	Not mapped CEGs	% complete*	Normalized (%)
Taxon A	Hybrid	218	30	87.9	95.2
	PacBio-only	227	21	91.5	99.1
Taxon B	Hybrid	217	29	87.5	94.8
	PacBio-only	225	23	90.7	98.3
Oryza sativa spp. *japonica*	Os-Nipponbare-Reference-IRGSP-1.0	229	19	92.3	100

*percentage of CEGs present
