# Supplementary material for: Sequencing of Australian wild rice genomes reveals ancestral relationships with domesticated rice
Source: Plant Biotechnol J. 2017 Jan 23;15(6):765–74. doi: 10.1111/pbi.12674 (PMC5425390; doi:10.1111/pbi.12674)
Supplement: Supplementary file 6 — Table S4 Completeness of Taxon A and Taxon B assemblies evaluated by means of presence of BUSCO orthologous genes. [file PBI-15-765-s002.pdf]

Table S4 Completeness of Taxon A and Taxon B assemblies evaluated by means of presence of BUSCO orthologous genes.

Taxa	Assembly	Single	Duplicated	Fragmented	Missing	% complete*	Normalized
Taxon A	Hybrid	832	246	46	78	87	89
	PacBio-only	918	212	22	16	96	98
Taxon B	Hybrid	830	255	56	70	86	89
	PacBio-only	836	170	55	65	87	89
Oryza sativa spp. *japonica*	Os-Nipponbare-Reference-IRGSP-1.0	936	216	10	10	97	100

*percentage of Complete Single-Copy BUSCOs present; Single – Complete Single-Copy BUSCOs; Duplicated – Complete Duplicated BUSCOs; Fragmented – Fragmented BUSCOs; Missing – Missing BUSCOs.
