# Supplementary material for: Sequencing of Australian wild rice genomes reveals ancestral relationships with domesticated rice
Source: Plant Biotechnol J. 2017 Jan 23;15(6):765–74. doi: 10.1111/pbi.12674 (PMC5425390; doi:10.1111/pbi.12674)
Supplement: Supplementary file 7 — Table S5 Unaligned and partially unaligned contig metrics of Australian wild rice taxa. [file PBI-15-765-s003.pdf]

Table S5 Unaligned and partially unaligned contig metrics of Australian wild rice taxa.

	**Taxon A**				**Taxon B**			
Reference genome	*O. sativa*		*O. meridionalis*		*O. sativa*		*O. meridionalis*	
Assembly	Hybrid	PacBio-only	Hybrid	PacBio-only	Hybrid	PacBio-only	Hybrid	PacBio-only
# unaligned contigs*	67	14	803	265	301	33	676	240
Fully unaligned length (Mb)	1.1	0.5	22.7	14.8	6.5	1.5	20.6	12.0
# partially unaligned contigs*	670	279	2,354	2,120	2,577	1,688	2,075	1,311
Partially unaligned length (Mb)	25.8	15.3	263.9	266.6	202.1	162.0	116.6	80.2
Unaligned length$^{\pm}$	26.9	15.9	286.6	281.3	208.6	163.5	137.3	92.3
Genome fraction (%)	70.7	71.5	23.6	25.4	42.4	37.3	62.4	56.3
# genes	38,587	33,346	4,981	5,402	26,532	22,588	18,006	16,357
# partial genes	8,106	10,063	8,950	9,421	9,399	8,794	8,822	7,805

*number of contigs without alignments to the reference sequences, fully or partially; $^{\pm}$total length of the unaligned contigs (sum of fully and partially unaligned regions in the assembly)
