# Supplementary material for: Sequencing of Australian wild rice genomes reveals ancestral relationships with domesticated rice
Source: Plant Biotechnol J. 2017 Jan 23;15(6):765–74. doi: 10.1111/pbi.12674 (PMC5425390; doi:10.1111/pbi.12674)
Supplement: Supplementary file 8 — Table S6 Length and GC content of Taxon A and Taxon B pseudomolecules and remaining contigs. [file PBI-15-765-s004.pdf]

Table S6 Length and GC content of Taxon A and Taxon B psuedosomocules and remaining contigs.

**Pseudomolecule**	**Taxon A**		**Taxon B**	
	%GC	Length	%GC	Length
Chr01	44.0%	41,912,295	43%	33,618,492
Chr02	43.8%	37,496,392	43%	27,986,264
Chr03	44.2%	39,112,528	43%	27,026,720
Chr04	44.1%	31,213,496	44%	31,144,760
Chr05	43.9%	29,753,236	43%	23,976,054
Chr06	43.8%	31,811,270	43%	22,865,571
Chr07	43.9%	28,478,632	43%	26,941,941
Chr08	43.6%	27,136,197	43%	22,577,491
Chr09	44.1%	23,188,492	43%	19,679,370
Chr10	43.8%	22,039,502	43%	18,408,352
Chr11	43.2%	27,446,056	43%	22,781,387
Chr12	43.2%	25,545,820	43%	17,848,930
Unordered	45.7%	19,846,994	43%	59,973,669
Total	43.9%	384,980,910	43%	354,829,001
