# Supplementary material for: Sequencing of Australian wild rice genomes reveals ancestral relationships with domesticated rice
Source: Plant Biotechnol J. 2017 Jan 23;15(6):765–74. doi: 10.1111/pbi.12674 (PMC5425390; doi:10.1111/pbi.12674)
Supplement: Supplementary file 9 — Table S7 Repetitive elements annotated in Taxon A genome. [file PBI-15-765-s005.pdf]

Table S7 Repetitive elements annotated in Taxon A genome.

		Genome coverage (bp)	Repeat #	Repeat fraction (%)	Genome fraction (%)
Class I (Retrotransposons)					
LTR	Copia	11,716,567	7,037	8.33	3.05
	Gypsy	81,228,038	27,133	57.77	21.11
	Retrovirus	609,338	182	0.43	0.16
	Other LTR	898,540	5,117	0.64	0.23
LINE	L1	4,284,820	5,478	3.05	1.11
	Other LINE	4,769	42	0.00	0.00
SINE	SINE	407,798	1,082	0.29	0.11
Other Class I		49,006	557	0.03	0.01
Class II (DNAt) subclass 1					
TIR	Tc1–Mariner	1,381,604	5,255	0.98	0.36
	hAT	2,183,755	3,465	1.55	0.57
	Mutator	8,417,944	9,364	5.99	2.19
	PIF–Harbinger	2,950,940	9,159	2.10	0.77
	CACTA	11,204,924	6,531	7.97	2.91
	Other DNAt	504,947	2,144	0.36	0.13
	MITE	1,715,292	5,719	1.22	0.45
Class II (DNAt) subclass 2					
Helitron		4,492,507	5,207	3.20	1.17
Other Class II		45,350	332	0.03	0.01
Total TEs		132,096,139	93,804	93.95	34.33
Ribosomal DNA		135,588	133	0.10	0.04
Structural Repeats		7,828,864	139,062	5.57	2.03
Unclassified		534,597	3,697	0.38	0.14
Total Repeats		140,595,188	236,696		36.54
