# Supplementary material for: Sequencing of Australian wild rice genomes reveals ancestral relationships with domesticated rice
Source: Plant Biotechnol J. 2017 Jan 23;15(6):765–74. doi: 10.1111/pbi.12674 (PMC5425390; doi:10.1111/pbi.12674)
Supplement: Supplementary file 10 — Table S8 Repetitive elements annotated in Taxon B genome. [file PBI-15-765-s006.pdf]

Table S8 Repetitive elements annotated in Taxon B genome.

		Genome coverage (bp)	Repeat #	Repeat fraction (%)	Genome fraction (%)
Class I (Retrotransposons)					
LTR	Copia	14,396,632	13,267	8.75	4.06
	Gypsy	65,230,139	37,092	39.65	18.38
	Retrovirus	367,124	263	0.22	0.10
	Other LTR	827,384	5,581	0.50	0.23
LINE	L1	4,842,831	9,820	2.94	1.36
	Other LINE	57,132	483	0.03	0.02
SINE	SINE	1,892,063	10,196	1.15	0.53
Other Class I		134,526	1,910	0.08	0.04
Class II (DNAt) subclass 1					
TIR	Tc1–Mariner	9,027,155	57,805	5.49	2.54
	hAT	4,338,182	17,762	2.64	1.22
	Mutator	15,222,784	50,417	9.25	4.29
	PIF–Harbinger	11,760,001	61,983	7.15	3.31
	CACTA	13,770,423	18,078	8.37	3.88
	Other DNAt	4,061,534	42,279	2.47	1.14
	MITE	5,690,040	45,488	3.46	1.60
Class II (DNAt) subclass 2					
Helitron		6,863,768	15,316	4.17	1.93
Other Class II		314,866	4,526	0.19	0.09
Total TEs		158,796,584	392,266	96.52	44.74
Ribosomal DNA		231,301	1,185	0.14	0.07
Structural Repeats		3,451,409	68,645	2.10	0.97
Unclassified		2,035,778	26,940	1.24	0.57
Total Repeats		164,515,072	489,036		46.35
