# Supplementary material for: Sequencing of Australian wild rice genomes reveals ancestral relationships with domesticated rice
Source: Plant Biotechnol J. 2017 Jan 23;15(6):765–74. doi: 10.1111/pbi.12674 (PMC5425390; doi:10.1111/pbi.12674)
Supplement: Supplementary file 11 — Table S9 Non‐coding RNA annotation in Taxon A and Taxon B genomes. [file PBI-15-765-s007.pdf]

**Table S9** Non-coding RNA annotation in Taxon A and Taxon B genomes.

| | Taxon A | | Taxon B | |
|---|---|---|---|---|
| | **number** | **length (bp)** | **number** | **length (bp)** |
| tRNAs | 677 | 50,687 | 615 | 46,301 |
| miRNA | 5,398 | 801,838 | 4,892 | 734,557 |
| snoRNA | 499 | 53,803 | 453 | 47,791 |
| sRNA | 91 | 13,717 | 87 | 13,341 |
| rRNA | 115 | 29,046 | 129 | 34,496 |
| Other* | 76 | 11,405 | 63 | 7,293 |
| Total | 6,179 | 909,809 | 5,624 | 837,478 |

tRNA – transfer RNA, miRNA – micro RNA, snoRNA – smll nucleolar RNA, sRNA – spliceosomal RNA, rRNA – ribosomal RNA

*other includes signal recognition particle RNA, nodulin 40, TPP riboswitch, iron stress repressed RNA, RNase MR
