# Supplementary material for: Sequencing of Australian wild rice genomes reveals ancestral relationships with domesticated rice
Source: Plant Biotechnol J. 2017 Jan 23;15(6):765–74. doi: 10.1111/pbi.12674 (PMC5425390; doi:10.1111/pbi.12674)
Supplement: Supplementary file 12 — Table S10 Protein coding genes annotation in Taxon A and Taxon B genomes. [file PBI-15-765-s008.pdf]

Table S10 Protein coding genes annotation in Taxon A and Taxon B genomes.

	**Taxon A**	**Taxon B**
# genes	22,035	21,169
# transcripts	27,473	26,735
# exons	99,618	97,975
# InterPro domain	13,505	13,072
# KEGG pathway	1,926	1,904
# GO annotation	8,763	8,466
