# Supplementary material for: Sequencing of Australian wild rice genomes reveals ancestral relationships with domesticated rice
Source: Plant Biotechnol J. 2017 Jan 23;15(6):765–74. doi: 10.1111/pbi.12674 (PMC5425390; doi:10.1111/pbi.12674)
Supplement: Supplementary file 13 — Table S11 Oryza and the outgroup species used in the phylogenetic study. [file PBI-15-765-s009.pdf]

Table S11 *Oryza* and the outgroup species used in the phylogenetic study.

Oryza species	Genome group	GenBank Assembly Accession
O. sativa japonica	AA	GCA_000005425.2
O. sativa indica	AA	GCA_000004655.2
O. rufipogon	AA	GCA_000817225.1
O. nivara	AA	GCA_000576065.1
O. barthii	AA	GCA_000182155.3
O. glaberrima	AA	GCA_000147395.2
O. glumaepatula	AA	GCA_000576495.1
O. meridionalis	AA	GCA_000338895.2
Taxon A	AA	LONB00000000
Taxon B	AA	LONC00000000
O. punctata	BB	GCA_000573905.1
O. brachyantha	FF	GCA_000231095.2
L. perrieri	–	GCA_000325765.3
