# Supplementary material for: Sequencing of Australian wild rice genomes reveals ancestral relationships with domesticated rice
Source: Plant Biotechnol J. 2017 Jan 23;15(6):765–74. doi: 10.1111/pbi.12674 (PMC5425390; doi:10.1111/pbi.12674)
Supplement: Supplementary file 14 — Table S12 The average rate of evolution estimates for Oryza species by chromosome. [file PBI-15-765-s010.pdf]

Table S12 The average rate of evolution estimates for *Oryza* species by chromosome.

Chromosome 1		Chromosome 2		Chromosome 3		Chromosome 4	
mean	stderr	mean	stderr	mean	stderr	mean	stderr
3.29E-03	1.82E-06	3.51E-03	1.76E-06	3.02E-03	1.53E-06	3.66E-03	1.90E-06

Chromosome 5		Chromosome 6		Chromosome 7		Chromosome 8	
mean	stderr	mean	stderr	mean	stderr	mean	stderr
3.78E-03	1.95E-06	3.38E-03	1.67E-06	3.83E-03	1.90E-06	3.79E-03	1.93E-06

Chromosome 9		Chromosome 10		Chromosome 11		Chromosome 12	
mean	stderr	mean	stderr	mean	stderr	mean	stderr
3.78E-03	2.04E-06	3.29E-03	1.89E-06	3.38E-03	1.78E-06	3.69E-03	2.00E-06
