# Supplementary material for: Sequencing of Australian wild rice genomes reveals ancestral relationships with domesticated rice
Source: Plant Biotechnol J. 2017 Jan 23;15(6):765–74. doi: 10.1111/pbi.12674 (PMC5425390; doi:10.1111/pbi.12674)
Supplement: Supplementary file 15 — Table S13 Divergence times estimates for Oryza species by chromosome. [file PBI-15-765-s011.pdf]

Table S13 Divergence times estimates for *Oryza* species by chromosome.

	Chromosome 1		Chromosome 2		Chromosome 3		Chromosome 4	
**Node**	**mya**	**95% HDP**	**mya**	**95% HDP**	**mya**	**95% HDP**	**mya**	**95% HDP**
A	14.98	14.04-15.96	14.97	14.07-16.01	14.99	14.04-16.02	14.98	14.07-16.05
B	6.72	6.26-7.14	6.86	6.41-7.31	6.80	6.35-7.25	6.76	6.32-7.23
C	2.66	2.48-2.83	3.17	2.92-3.38	2.79	2.61-2.99	3.16	2.95-3.37
D	2.02	1.89-2.15	2.11	1.97-2.25	2.07	1.93-2.21	1.50	1.39-1.60
E	1.57	1.47-1.68	1.71	1.59-1.82	1.40	1.31-1.50	1.73	1.62-1.85
F	1.14	1.06-1.23	1.33	1.24-1.42	1.16	1.09-1.24	1.24	1.15-1.32
G	1.02	0.95-1.09	1.14	1.07-1.22	0.99	0.92-1.06	1.06	0.99-1.13
H	0.91	0.84-0.98	1.09	1.02-1.17	0.97	0.90-1.04	0.96	0.89-1.03
I	0.68	0.63-0.73	0.82	0.76-0.88	0.75	0.69-0.80	0.78	0.73-0.84
J	0.59	0.55-0.64	0.62	0.58-0.66	0.60	0.56-0.65	0.60	0.55-0.64
K	0.51	0.48-0.55	0.72	0.67-0.77	0.49	0.46-0.53	0.63	0.59-0.68

	Chromosome 6		Chromosome 8		Chromosome 9		Chromosome 12	
**Node**	**mya**	**95% HDP**	**mya**	**95% HDP**	**mya**	**95% HDP**	**mya**	**95% HDP**
A	14.98	14.01-15.91	14.99	14.05-15.95	14.99	14.03-15.96	14.98	14.04-16.01
B	6.86	6.39-7.29	6.77	6.34-7.22	6.88	6.43-7.35	6.59	6.13-7.03
C	2.96	2.77-3.16	3.09	2.88-3.30	3.09	2.88-3.30	2.93	2.73-3.14
D	1.61	1.50-1.72	1.08	1.00-1.16	1.48	1.37-1.59	1.72	1.59-1.85
E	1.60	1.48-1.70	1.74	1.62-1.86	1.60	1.49-1.72	1.90	1.77-2.04
F	1.24	1.16-1.32	1.30	1.20-1.38	1.25	1.16-1.33	1.55	1.44-1.66
G	1.04	0.97-1.11	1.20	1.11-1.28	1.18	1.09-1.26	1.19	1.10-1.28
H	0.96	0.89-1.02	0.94	0.87-1.01	1.07	0.99-1.15	1.00	0.93-1.08
I	0.75	0.69-0.80	0.77	0.71-0.82	0.72	0.67-0.78	0.74	0.68-0.80
J	0.59	0.54-0.63	0.54	0.50-0.59	0.80	0.74-0.86	0.46	0.42-0.51
K	0.58	0.54-0.62	0.76	0.71-0.82	0.84	0.78-0.90	0.81	0.74-0.87

Chromosomes with different tree topology than the majority of chromosomes

	Chromosome 5		Chromosome 7		Chromosome 10		Chromosome 11	
**Node**	**mya**	**95% HDP**	**mya**	**95% HDP**	**mya**	**95% HDP**	**mya**	**95% HDP**
A	14.98	14.00-15.95	14.99	14.08-16.00	14.99	14.00-15.99	14.99	14.01-15.96
B	6.72	6.29-7.17	6.71	6.27-7.15	6.12	5.67-6.58	7.09	6.61-7.60
C	3.13	2.93-3.34	2.76	2.57-2.94	2.02	1.85-2.19	2.39	2.21-2.57

D	1.66	1.55-1.78	0.96	0.90-1.03	0.62	0.55-0.70	1.25	1.13-1.36
E	1.63	1.52-1.74	1.57	1.47-1.68	0.92	0.84-1.00	1.64	1.51-1.76
F	1.35	1.26-1.44	1.18	1.10-1.26	0.77	0.70-0.84	1.53	1.42-1.65
G	1.44	1.35-1.54	1.38	1.29-1.47	0.79	0.72-0.87	1.27	1.17-1.37
H	1.01	0.94-1.08	0.92	0.86-0.99	0.71	0.65-0.78	0.94	0.87-1.03
I	0.8	0.74-0.85	0.77	0.71-0.82	0.55	0.49-0.61	0.69	0.62-0.75
J	0.58	0.53-0.62	0.68	0.63-0.73	0.34	0.29-0.40	0.46	0.41-0.52
K	0.92	0.66-0.99	0.66	0.61-0.70	0.24	0.20-0.28	0.51	0.45-0.57
