# Supplementary material for: Sequencing of Australian wild rice genomes reveals ancestral relationships with domesticated rice
Source: Plant Biotechnol J. 2017 Jan 23;15(6):765–74. doi: 10.1111/pbi.12674 (PMC5425390; doi:10.1111/pbi.12674)
Supplement: Supplementary file 16 — Table S14 Global statistics calculated in four‐taxon test for selected Oryza species per chromosome. [file PBI-15-765-s012.pdf]

Table S14 Global statistics calculated in four-taxon test for selected *Oryza* species per chromosome.

Four taxon test for a set (((*O. rufipogon, O. barthii*), Taxon A), *O. punctata*)

	**Chr 1**	**Chr 2**	**Chr 3**	**Chr 4**	**Chr 5**	**Chr 6**
Base pairs	885,116	996,507	805,108	706,297	658,924	551,896
Polymorphic sites	71,201	96,385	61,486	74,967	70,350	52,933
ABBA	1,278	2,853	772	2,018	1,449	1,096
BABA	1,787	3,341	1,888	2,275	2,872	1,813
D-statistic	-0.16607	-0.07879	-0.41955	-0.05986	-0.32932	-0.2465
Z-score	-1.9633	-0.72932	-3.74382	-0.6485	-3.42562	-1.9795

	**Chr 7**	**Chr 8**	**Chr 9**	**Chr 10**	**Chr 11**	**Chr 12**
Base pairs	533,899	350,596	345,315	93087	134,216	211,890
Polymorphic sites	55,811	41,850	36,600	5588	12,562	19,252
ABBA	1,781	313	783	206	133	244
BABA	972	1,580	1,557	186	288	822
D-statistic	0.29386	-0.66931	-0.33077	0.05102	-0.36817	-0.5422
Z-score	2.55576	-10.03458	-2.85748	0.1037	-2.51294	-3.6627

Four taxon test for a set (((*O. rufipogon, O. nivara*), Taxon A), *O. punctata*)

	**Chr 1**	**Chr 2**	**Chr 3**	**Chr 4**	**Chr 5**	**Chr 6**
Base pairs	885,116	996,507	805108	706,297	658924	551896
Polymorphic sites	68,110	90,534	57,274	70,570	64,356	50,122
ABBA	1,638	2,363	852	1,821	1,319	1,227
BABA	957	1,417	1,090	848	2,012	1,028
D-statistic	0.26243	0.25026	-0.12255	0.36456	-0.20805	0.08825
Z-score	2.58619	2.37828	-0.99487	2.85723	-1.79152	0.56591

	**Chr 7**	**Chr 8**	**Chr 9**	**Chr 10**	**Chr 11**	**Chr 12**
Base pairs	533899	350596	345315	93087	134216	211,890
Polymorphic sites	51,798	37,595	33,296	5,682	11,661	18,963
ABBA	1,595	761	581	53	91	299
BABA	1,136	595	696	79	119	184
D-statistic	0.16807	0.12242	-0.09005	-0.19697	-0.13333	0.23810
Z-score	1.14980	0.66700	-0.74602	-0.50227	-1.51219	2.73644

Columns marked in green and red indicate significant Z-score for negative and positive D-statistic,

respectively.
