# Supplementary material for: Sequencing of Australian wild rice genomes reveals ancestral relationships with domesticated rice
Source: Plant Biotechnol J. 2017 Jan 23;15(6):765–74. doi: 10.1111/pbi.12674 (PMC5425390; doi:10.1111/pbi.12674)
Supplement: Supplementary file 17 — Table S15 Summary of data used in divergence time estimation. [file PBI-15-765-s013.pdf]

Table S15 Summary of data used in divergence time estimation.

Chromosome	# genes	Alignment length
1	659	885,116
2	748	996,507
3	593	805,108
4	535	706,297
5	511	658,924
6	398	551,896
7	372	533,899
8	272	350,596
9	256	345,315
10	65	93,087
11	98	134,216
12	136	211,890
Total	4,643	6,272,851
